# Supplementary material for: Social Media Usage and Advertising Food-Related Content: Influence on Dietary Choices of Gen Z
Source: Nutrients. 2025 Dec 16;17(24):3930. doi: 10.3390/nu17243930 (PMC12735408; doi:10.3390/nu17243930)
Supplement: Supplementary file 1 [file nutrients-17-03930-s001.zip › Supplementary file S1.pdf]

Impact of Social Media Marketing (Digital marketing) and Food-Related Content on dietary choices of young adults (18-25) from Surat, Gujarat

Dear Participants,
This research is being undertaken for partial fulfilment of M.Sc. Nutrition and Dietetics programme at Symbiosis International Deemed University.
Title: Impact of Social Media Marketing (Digital marketing) and Food-Related Content on dietary choices of young adults (18-25)

Purpose of the study: In this study, we will try to understand how To Measure The Impact Of Social Media Marketing (Digital marketing) And Food Related Content On Dietary choices of young adults (18-25). The main objective of this study is to determine the impact of Social Media (SM) marketing of Food Ads and Content on Dietary choices of young adults (18-25).

Proposed activities: If you agree to participate in this study, you will be asked questions about your social media content consumption pattern and dietary habits. Your weight and height will also be recorded. You will have to answer those questions. While answering some questions, you will have to pick one of the appropriate options.
This may take a time period of 15 to 20 minutes.

By participating in this research study, there is no possibility of any potential risk to you / There are no additional risks involved. Your study records will be kept confidential and would be used only for the purpose of research. Nobody outside the research team will have access to the information without your written authorization. Study information will be made available by the research team only to the authorized personnel involved in the study and the ethics committee.

If you have any queries about this study and about your rights as a research participant in this study, or if you do not feel to provide any information about you; you may contact the Principal Investigator of this study at any time. The contact information of the Principal Investigator is provided below:

RASHI NANDWANI
EMAIL- rashi.nandwanimscnd2022@sihspune.org

* Required

Skip to question 1Skip to question 1

CONSENT FORM

1. The details of the study have been provided to me in writing, and I have read all the information provided to me (Or the details of the study have been explained to me).
2. I had opportunity to ask the questions or any doubts; and my all queries have been answered to my satisfaction, and all the doubts have been resolved.
3. I understand that my participation in the study is voluntary, and that I am free to withdraw at any time, without giving any reason, without the benefit / medical care being affected or any of my legal rights being affected.
4. I agree not to restrict the use of any data or results that arise from this study, provided such a use is only for scientific purpose(s). I agree to take part in the above study.

1. I have fully understood the details of this study as described above and voluntarily agree to participate

Mark only one oval.

- [ ] Yes, I agree
- [ ] No, I disagree

BACKGROUND INFORMATION

This section collects your background information.

2. Email

____

3. Name: *

____

4. Sex *

Mark only one oval.

- Male
- Female
- Prefer not to say
- Other

5. Age (18-25) *

6. Occupation *

7. Are you currently pregnant/ lactating (breastfeeding)? *

Mark only one oval.

- Yes
- No
- Not applicable

8. Height. (For example 5'2") *

9. Weight (kg) *

10. What is the highest degree or level of education you have completed? *

Mark only one oval.

- Bachelors Degree
- Masters degree
- Class 12
- PhD

11. Are you diagnosed with any of the following medical conditions?

Check all that apply.

- Diabetes(Type 1/Type 2)
- Hypertension (high blood pressure)
- PCOS
- Kidney Disease
- Respiratory Disaese
- NONE OF THE ABOVE
- Other: ____________________

DIETARY HABITS

This section assesses your social media usage trends.

12. How many meals do you consume in a day? *

Mark only one oval.

- 2
- 3
- 4
- 5
- Other: ____________________

13. Do you have a habit of consuming breakfast regularly? *

Mark only one oval.

- Yes
- No
- Sometimes
- Always

14. Do you feel hungry other than at mealtimes? *

Mark only one oval.

- Yes
- No
- Sometimes
- Always

15. Do you follow food content creators and social media influencers sharing food-related content? *

Mark only one oval.

- Yes
- No

16. Have you ever bought or ordered a food product that was showcased in a food Ad or content on social media? *

Mark only one oval.

- Yes
- No

17. When you see a food Ad or content, do you often get so hungry that you want to make or order it right away? *

Mark only one oval.

- Yes
- No

18. When buying a food product or ordering something, which of the following do you get influenced by? *

Check all that apply.

- Advertisement
- Offers/Discount
- Mood
- Hunger
- Budget

SOCIAL MEDIA USAGE

This section assesses your social media usage trends.

19. How many times a day do you look at social media? *

Mark only one oval.

- Not everyday
- Once a day
- 2-5 times a day
- 5-10 times a day
- Other: __________

20. How much time do you spend on social media per day? *

Mark only one oval.

- 1- 2 hours
- 2-3 hours
- 3 hours +
- Other: ____________

21. When do you access social media? *

Check all that apply.

- During free time
- Whilst at college/ work
- During social occasions
- Mealtimes
- During Commute

22. Which social media sites have you watched food ads and food content on? *

Check all that apply.

- YouTube
- Facebook
- Pinterest
- Snapchat
- Instagram
- Twitter
- NONE OF THE ABOVE
- Other: ____________

23. Are you attracted to a food brand by watching the advertisement? *

Mark only one oval.

- Yes
- No
- Sometimes

24. Which of the following Food Ads do you come across on your social media. *

Check all that apply.

- [ ] Pizza hut
- [ ] McDonald's
- [ ] Domino's
- [ ] Burger King
- [ ] Subway
- [ ] Zomato
- [ ] Swiggy
- [ ] Maggi
- [ ] Cadbury silk
- [ ] Snickers
- [ ] KitKat
- [ ] KFC
- [ ] NONE OF THE ABOVE
- [ ] Other: ____________________

25. Do you follow any of these food Influencers and bloggers? If yes, select the ones whose content you have watched? *

Check all that apply.

- [ ] Bake with shivesh
- [ ] Chahat Anand
- [ ] Zingy Zest
- [ ] Dil se foodie
- [ ] So sauté
- [ ] Priyanka Tiwari
- [ ] Parth Bajaj
- [ ] Archit Agarwal (@architlost)
- [ ] HungryDilliWali
- [ ] Pooja Dhingra
- [ ] Saransh Goila
- [ ] Natasha Gandhi
- [ ] Chef Ranveer Brar
- [ ] Kabitas Kitchen
- [ ] Kanaks Kitchen
- [ ] Saloni Kukreja
- [ ] Anahita Dhondhy
- [ ] NONE OF THE ABOVE
- [ ] Other: ____________________

26. How often do you see food content and ads on the social media you use? *

Mark only one oval.

- ( ) After every few minutes
- ( ) After every few posts and stories
- ( ) Every day
- ( ) Once a week
- ( ) NEVER

27. Do you love watching food ads and content? *

Mark only one oval.

- Yes
- No

28. How likely are you to try a food/food product/Restaurant/ Eatery promoted by a social media influencer? *

Mark only one oval.

- Very Frequently
- Frequently
- Occasionaly
- Rarely
- Never

29. Do you like to view food pictures others post on social media? *

Mark only one oval.

- Yes
- No
- Sometimes

30. Do you order and eat something you see in a food ad/content when you are stressed/emotional?

Mark only one oval.

- Yes
- No

HUNGER SCALE

In the questions below is a simple scale for measuring the amount of feeling of hunger that is generated after seeing the below-presented food images. The participant is simply needed to mark the degree that resembles the amount of hunger. The lower end of the scale 0-4 represents Low hunger, 5-7 represents moderate hunger and 8-10 represents high hunger.

31. On a scale of 1-10 How Hungry do you feel after looking at this picture ? *

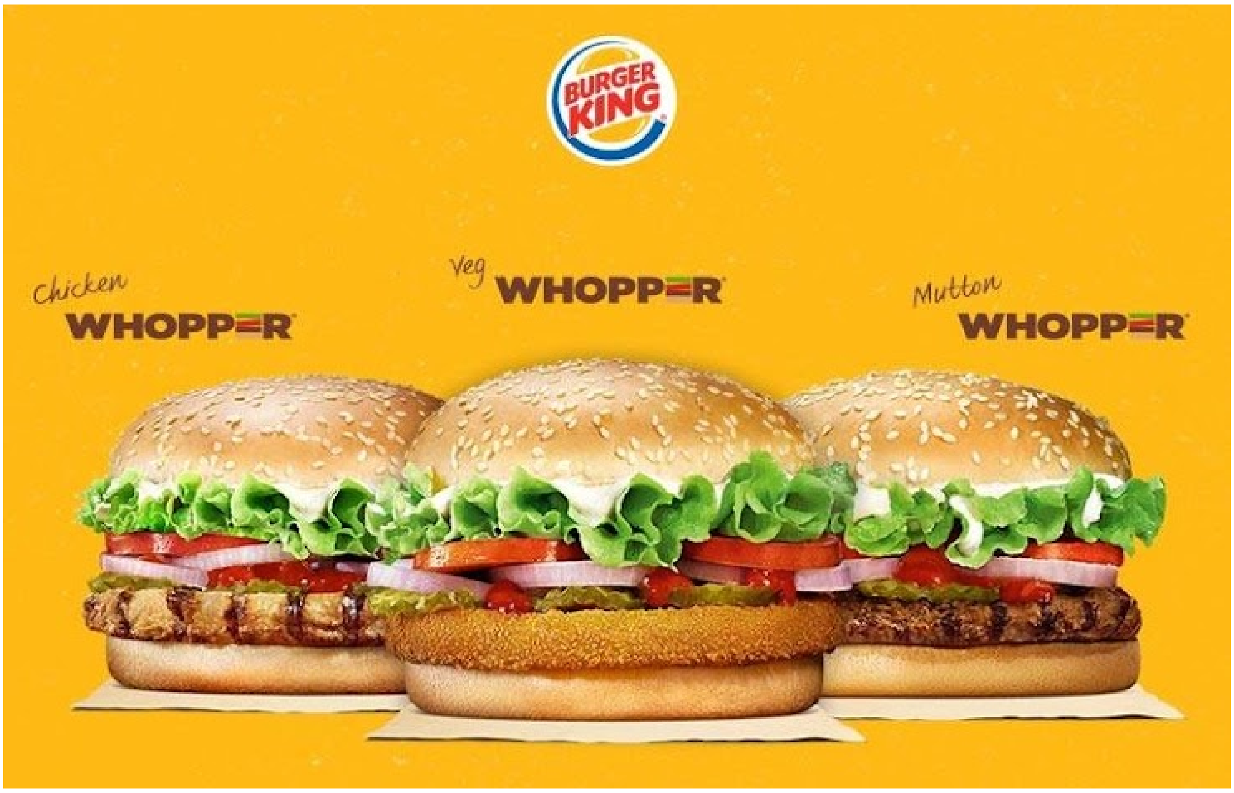

Mark only one oval.

1	2	3	4	5	6	7	8	9	10
⬭	⬭	⬭	⬭	⬭	⬭	⬭	⬭	⬭	⬭

32. On a scale of 1-10 How Hungry do you feel after looking at this picture ? *

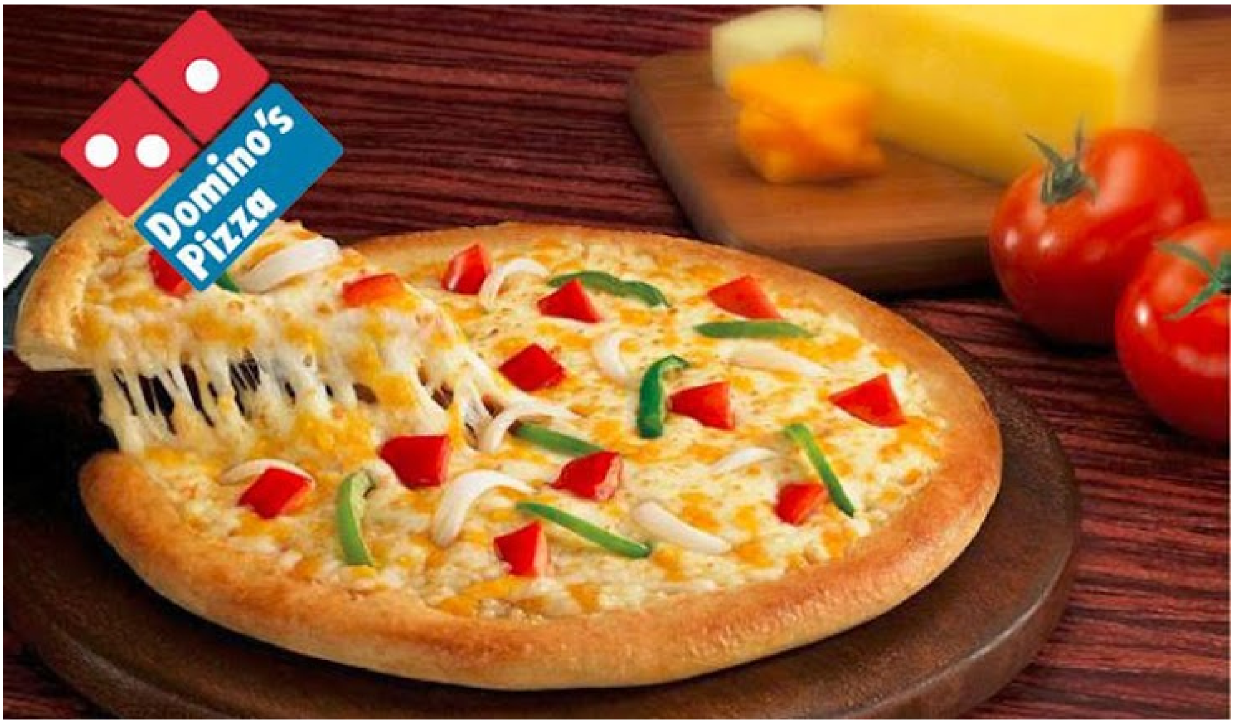

Mark only one oval.

1	2	3	4	5	6	7	8	9	10
◯	◯	◯	◯	◯	◯	◯	◯	◯	◯

33. On a scale of 1-10 How Hungry do you feel after looking at this picture ? *

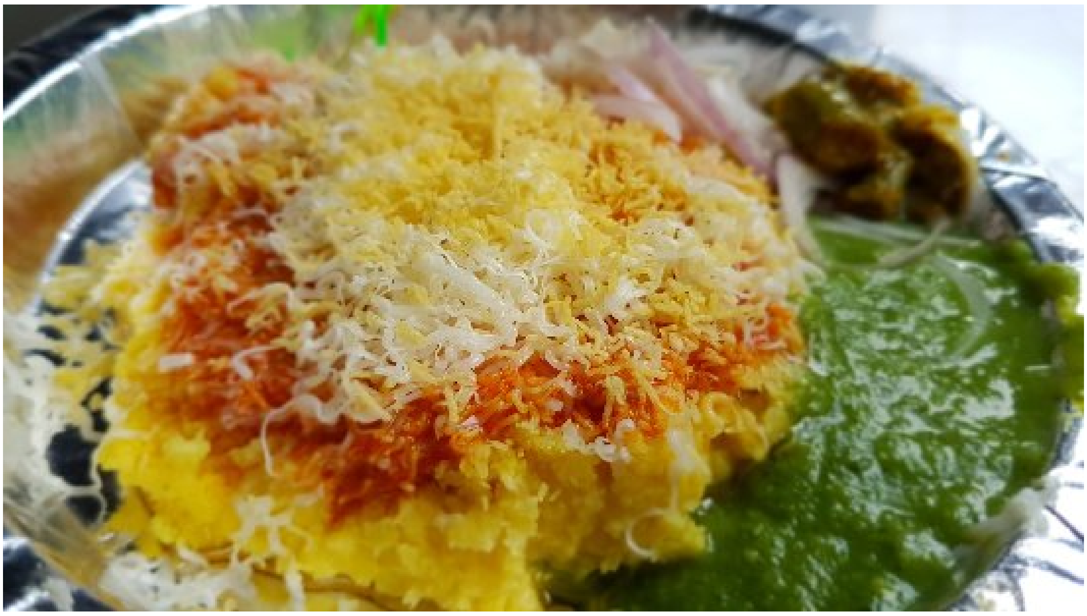

Mark only one oval.

1	2	3	4	5	6	7	8	9	10
◯	◯	◯	◯	◯	◯	◯	◯	◯	◯

34. On a scale of 1-10 How Hungry do you feel after looking at this picture ? *

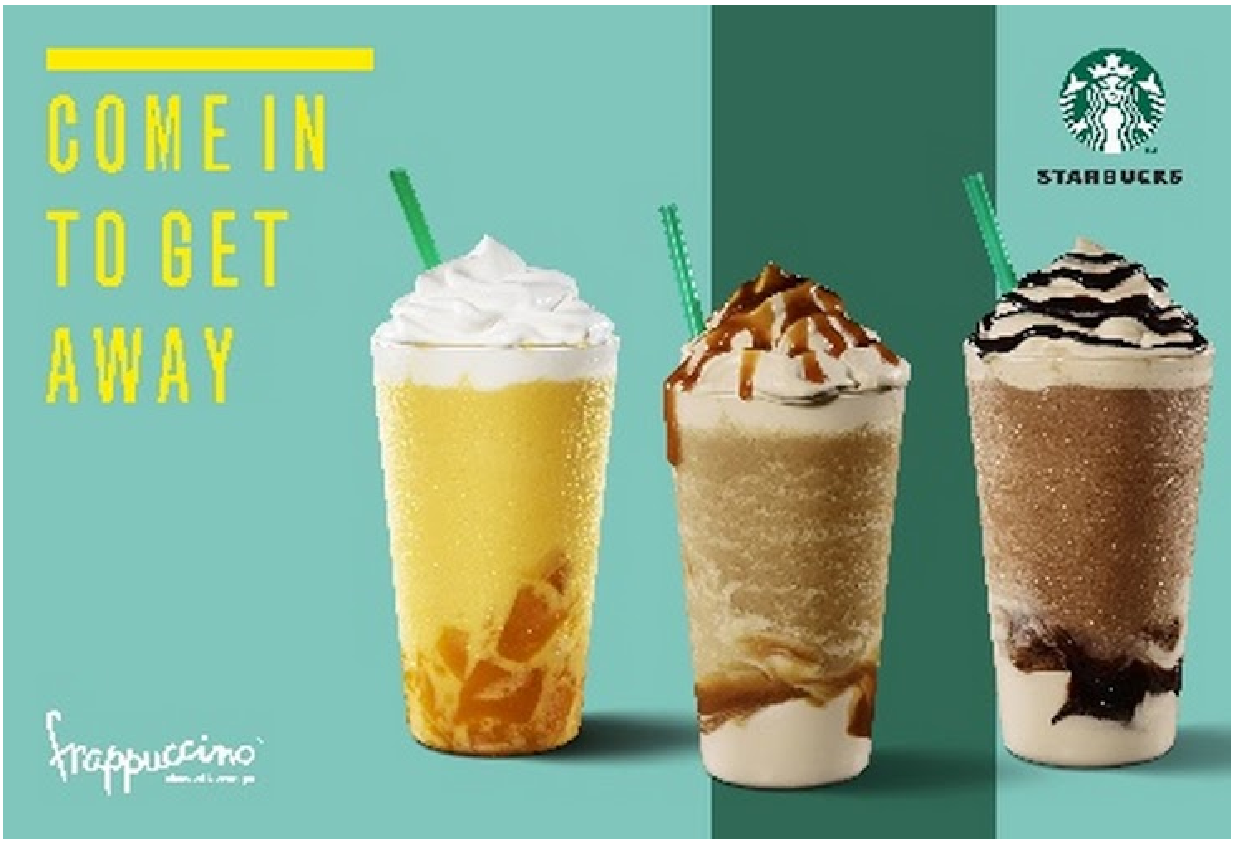

Mark only one oval.

1	2	3	4	5	6	7	8	9	10
◯	◯	◯	◯	◯	◯	◯	◯	◯	◯

35. On a scale of 1-10 How Hungry do you feel after looking at this picture ?

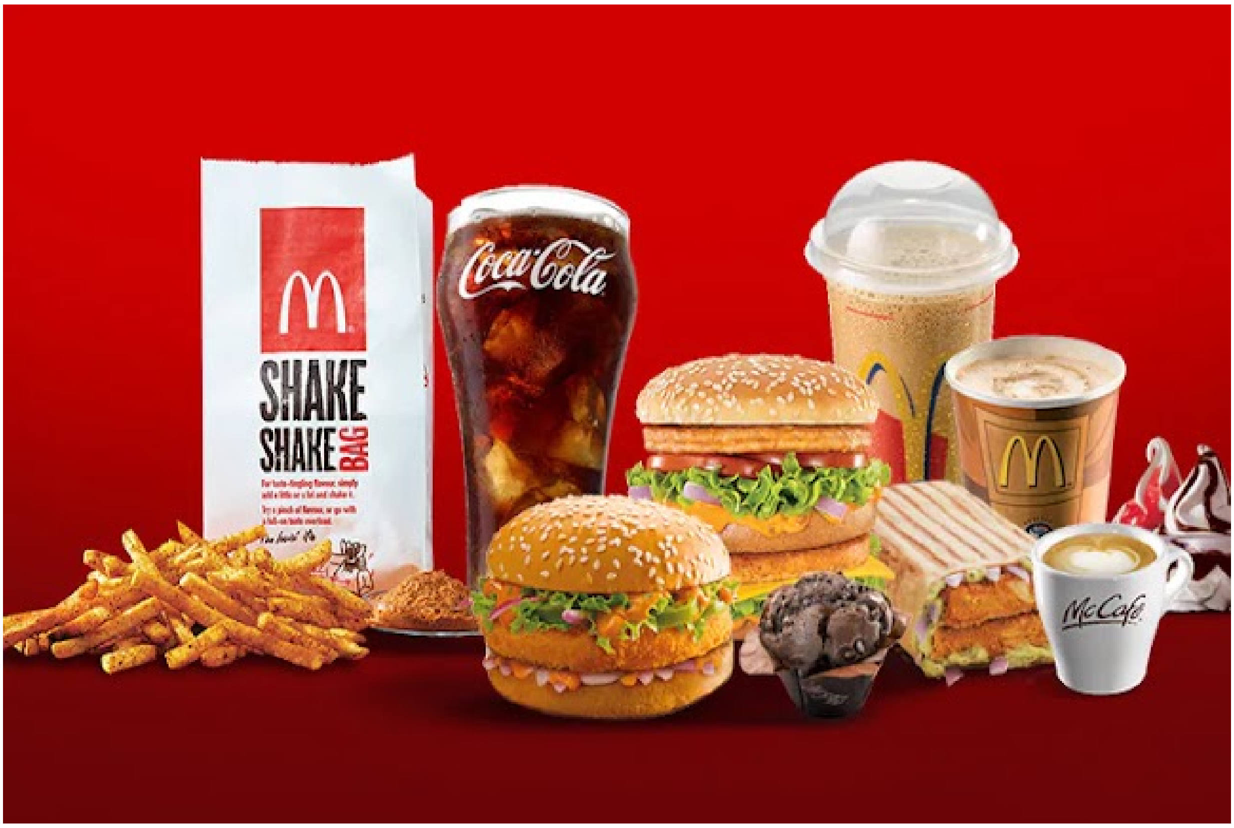

Mark only one oval.

1	2	3	4	5	6	7	8	9	10
⬭	⬭	⬭	⬭	⬭	⬭	⬭	⬭	⬭	⬭

36. On a scale of 1-10 How Hungry do you feel after looking at this picture ? *

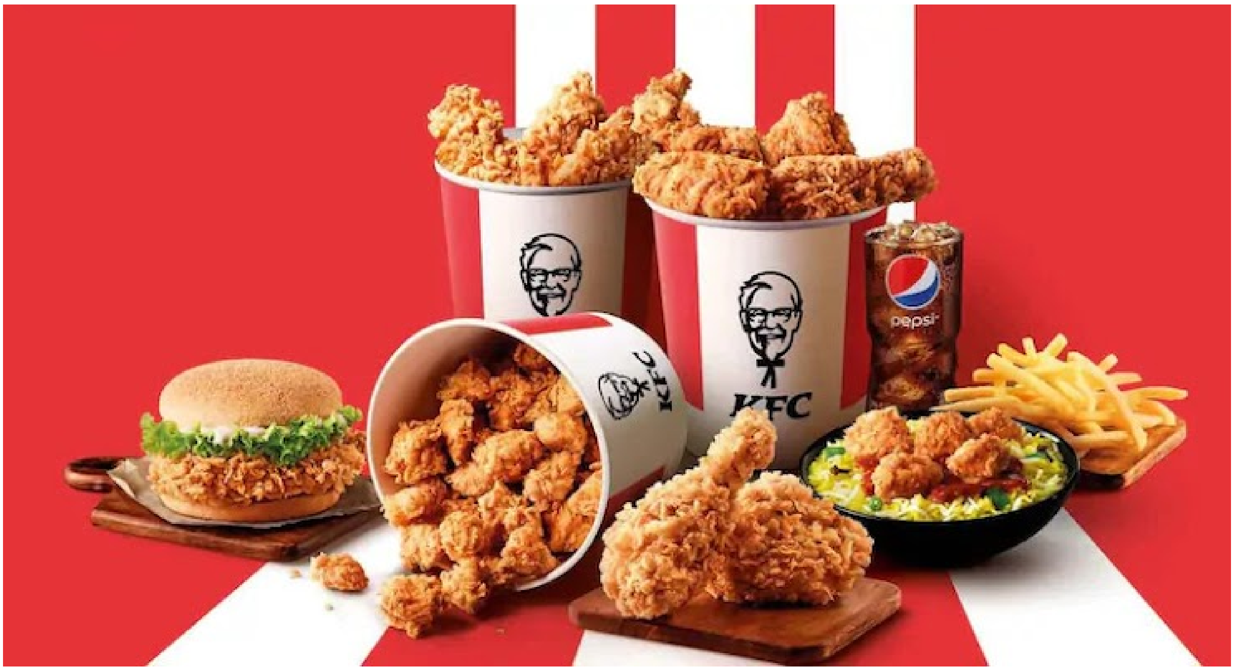

Mark only one oval.

1	2	3	4	5	6	7	8	9	10
⬭	⬭	⬭	⬭	⬭	⬭	⬭	⬭	⬭	⬭

37. On a scale of 1-10 How Hungry do you feel after looking at this picture ? *

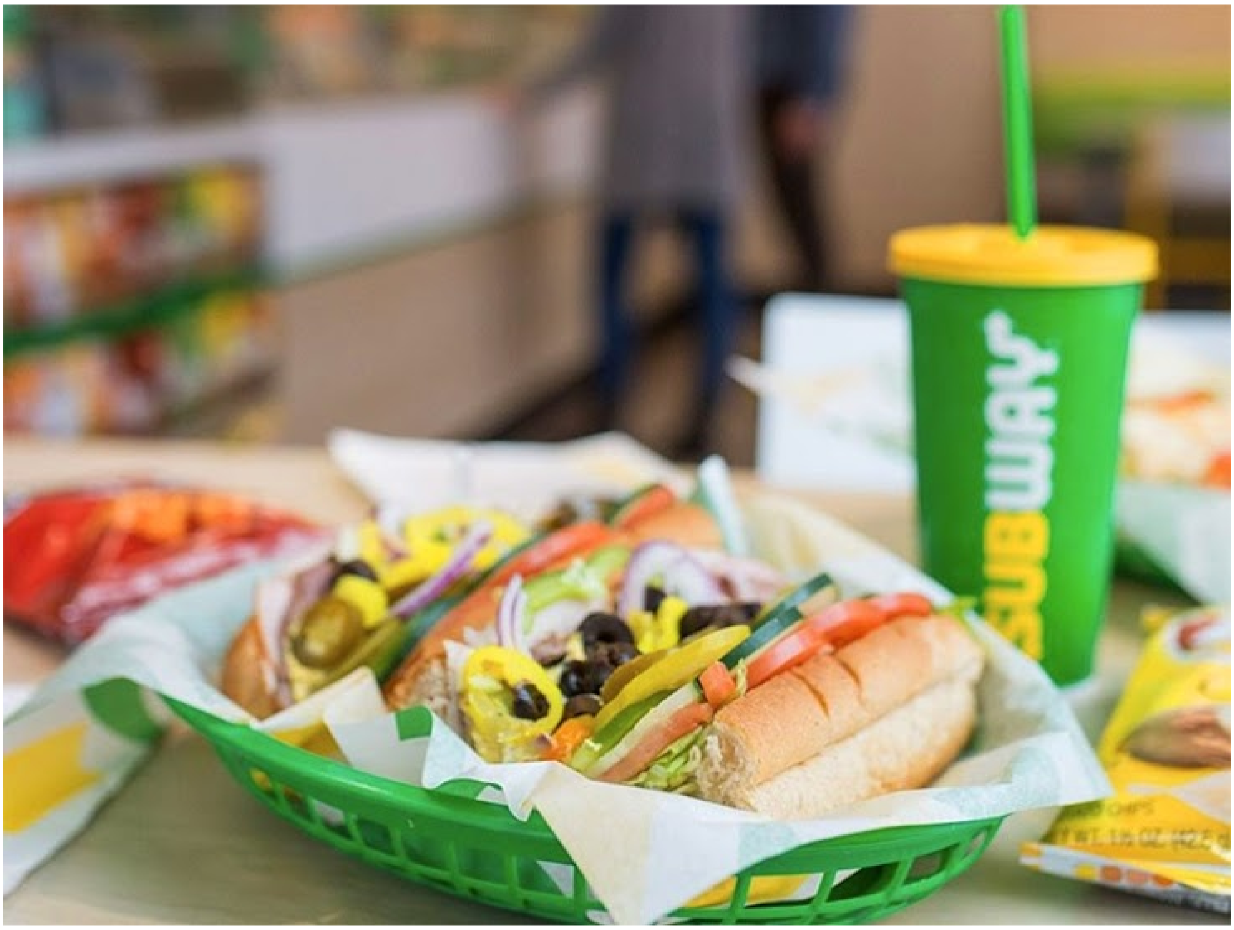

Mark only one oval.

1	2	3	4	5	6	7	8	9	10
◯	◯	◯	◯	◯	◯	◯	◯	◯	◯

38. On a scale of 1-10 How Hungry do you feel after looking at this picture ? *

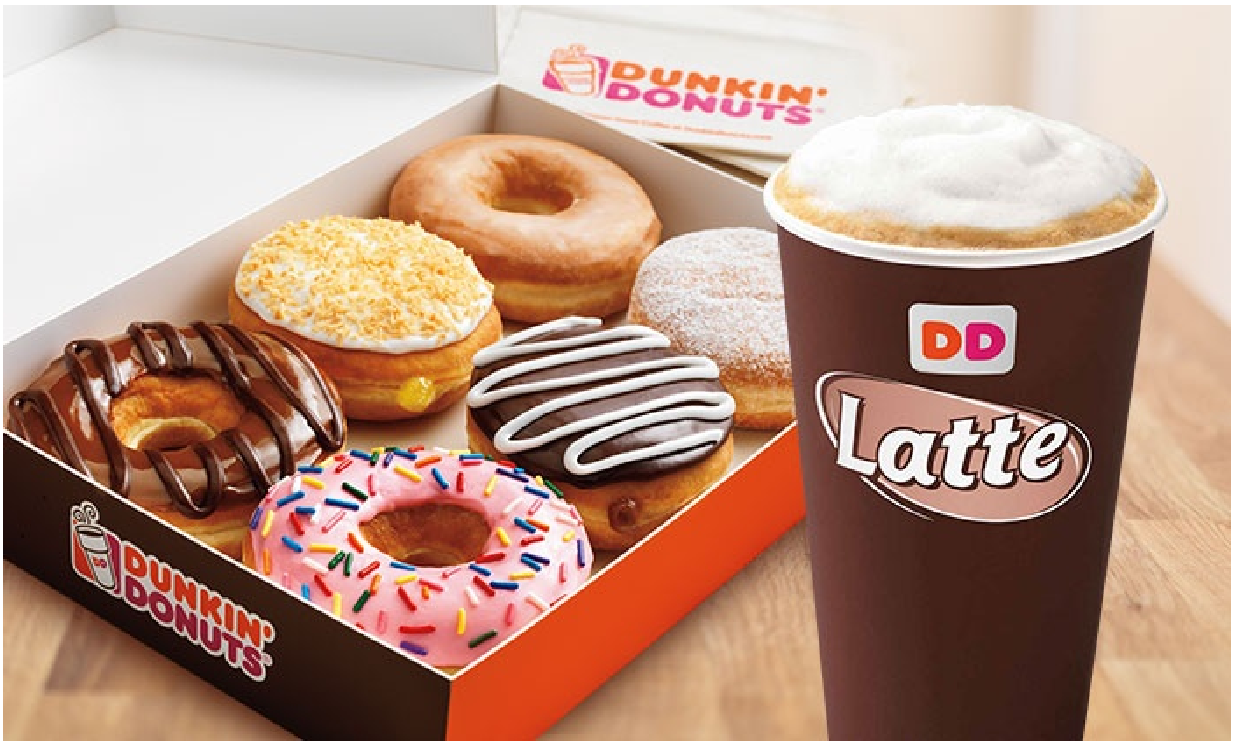

Mark only one oval.

1	2	3	4	5	6	7	8	9	10
⬭	⬭	⬭	⬭	⬭	⬭	⬭	⬭	⬭	⬭

39. On a scale of 1-10 How Hungry do you feel after looking at this picture ? *

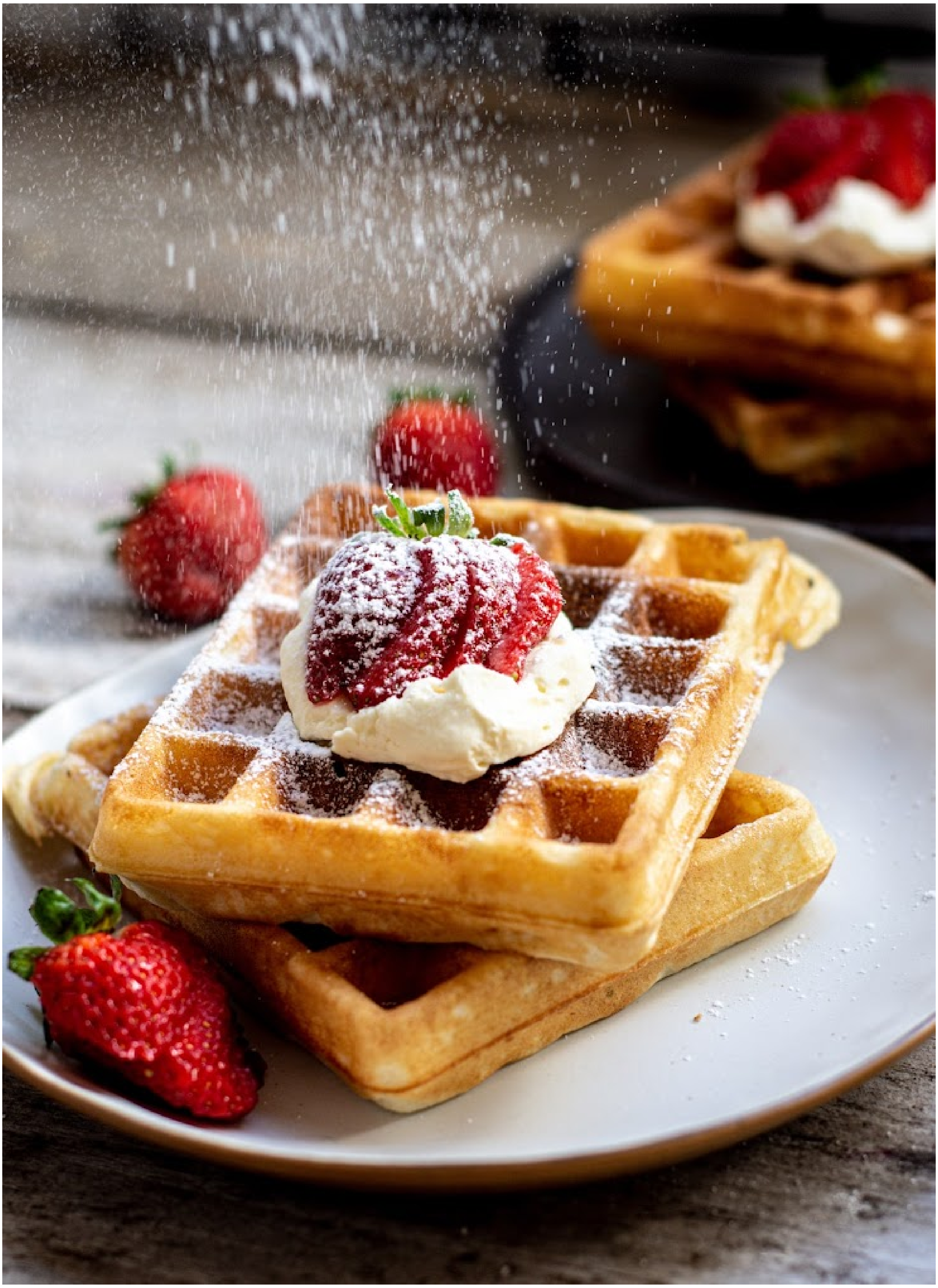

Mark only one oval.

1	2	3	4	5	6	7	8	9	10
◯	◯	◯	◯	◯	◯	◯	◯	◯	◯

40. On a scale of 1-10 How Hungry do you feel after looking at this picture ? *

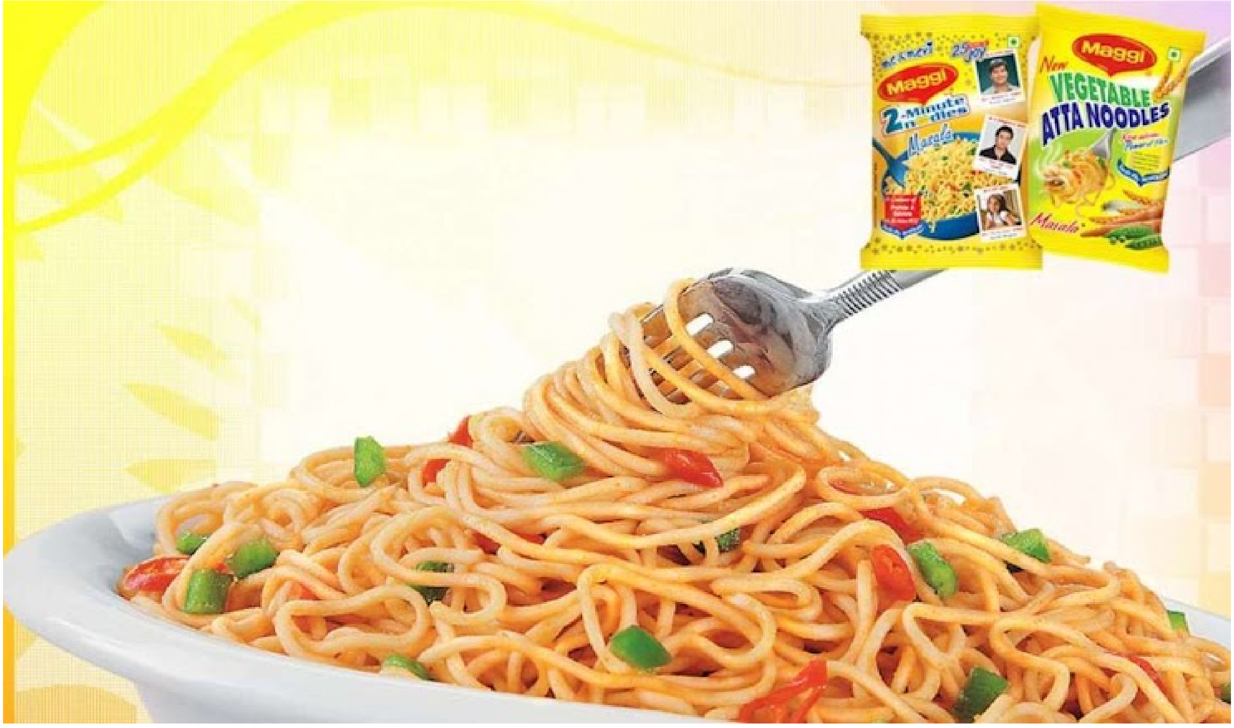

Mark only one oval.

	1	2	3	4	5	6	7	8	9	10	

41. On a scale of 1-10 How Hungry do you feel after looking at this picture ?

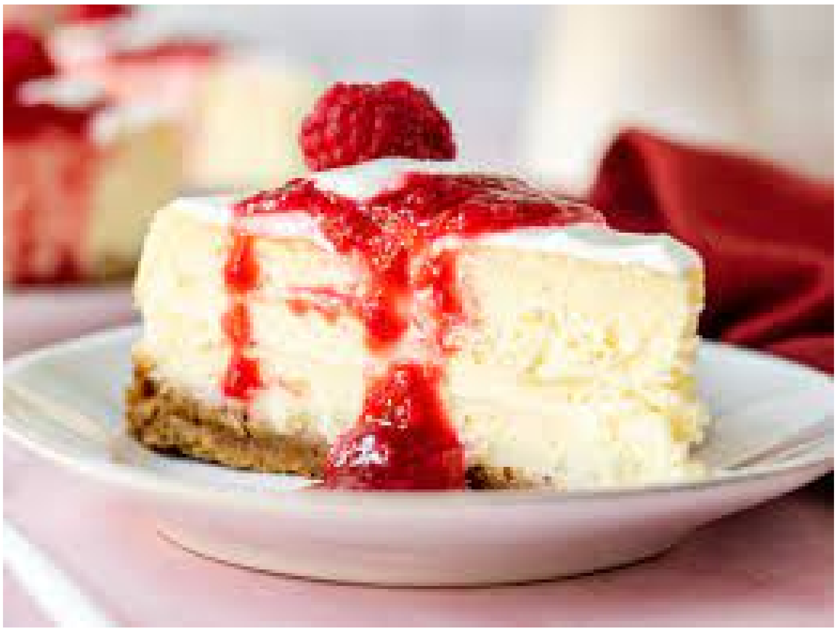

Mark only one oval.

1	2	3	4	5	6	7	8	9	10
◯	◯	◯	◯	◯	◯	◯	◯	◯	◯

42. On a scale of 1-10 How Hungry do you feel after looking at this picture ?

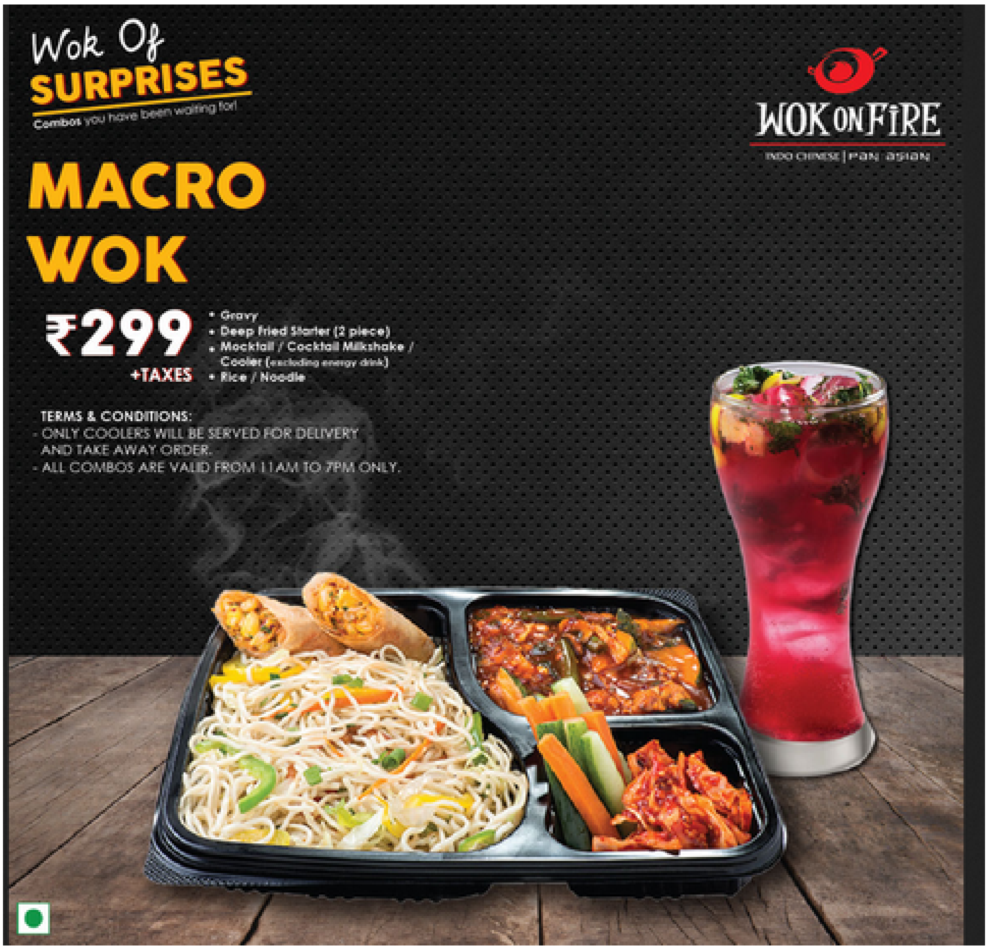

Mark only one oval.

1	2	3	4	5	6	7	8	9	10
⬭	⬭	⬭	⬭	⬭	⬭	⬭	⬭	⬭	⬭

Thank you

Thank you for your participation in my study. For any further queries regarding this research, please feel to reach out at rashi.nandwanimscnd2022@sihspune.org

This content is neither created nor endorsed by Google.

Google Forms
