# Supplementary material for: Social Media Usage and Advertising Food-Related Content: Influence on Dietary Choices of Gen Z
Source: Nutrients. 2025 Dec 16;17(24):3930. doi: 10.3390/nu17243930 (PMC12735408; doi:10.3390/nu17243930)
Supplement: Supplementary file 1 [file nutrients-17-03930-s001.zip › Supplementary file S2.pdf]

# CODES FOR QUESTIONNAIRE FOR DATA ANALYSIS

## Background Information

1. SEX
   1- Male
   2- Female
2. AGE
   1- < 20(MEAN)
   2- ≥ 20
3. BMI
   1- Underweight
   2- Normal
   3- Overweight, Pre-obese and obese
4. Highest Level of education attained
   1- Class 12
   2- Bachelor's Degree
   3- Masters Degree
5. Medical Conditions
   0-None of the above
   1-Hyperthyroidism
   2-Kidney disease
   3-Migraine
   4-PCOS
   5-Respiratory disease

## Meal Information

6. Meal consumed in a day
   1- 1 meal
   2- 2 meal
   3- 3 meal
   4- 4 and 4+ meal
7. Breakfast consumed
   0- No
   1- Yes
   2- Always
   3- Sometimes
8. Do you feel hungry other than mealtimes
   0- No
   1- Yes
   2- Always
   3- Sometimes
9. Follow food content creators?
   0- No
   1- Yes
10. Bough food product showcased in an Ad

    1- No

2- Yes
11. Order right away after seeing an Ad
    0- No
    1- Yes
12.

## Social Media Usage

13. How many times a day you look at social media?
    0- Not everyday
    1- Once a day,
    2- 2-5 times a day ,
    3- 5-10 times a day
    4- More than 10 times a day
14. How much time you spend on social media?
    1- 1- 2 hours
    2- 2-3 hours
    3- 3+hours
15. When you access social media
    1- During free time
    2- Whilst at college/ work
    3- During social occasions
    4- Mealtimes
    5- During Commute
    6- During free time, During Commute
    7- During free time, Mealtimes
    8- During free time, Whilst at college/ work
    9- During free time, During social occasions
    10- During free time, During social occasions, During Commute
    11- During free time, During social occasions, Mealtimes
    12- During free time, Mealtimes, During Commute
    13- During free time, Whilst at college/ work, During social occasions
    14- During free time, Whilst at college/ work, Mealtimes
    15- Whilst at college/ work, During social occasions, During Commute
    16- During free time, Whilst at college/ work, Mealtimes, During Commute
    17- During free time, Whilst at college/ work, During social occasions, Mealtimes
    18- During free time, Whilst at college/ work, During social occasions, Mealtimes, During Commute
16. Social media sites
    0- NONE OF THE ABOVE
    1- YouTube
    2- Facebook
    3- Pinterest
    4- Snapchat
    5- Instagram
    6- Twitter
    7- YouTube, Instagram
    8- Snapchat, Instagram

9- YouTube, Pinterest
10- Facebook, Instagram
11- Pinterest, Instagram
12- YouTube, Facebook
13- YouTube, Snapchat
14- Pinterest, Snapchat, Instagram
15- YouTube, Facebook, Snapchat
16- YouTube, Snapchat, Instagram
17- YouTube, Instagram, Twitter
18- YouTube, Facebook, Instagram
19- YouTube, Pinterest, Instagram
20- YouTube, Pinterest, Snapchat, Instagram
21- YouTube, Facebook, Snapchat, Instagram
22- YouTube, Facebook, Instagram, Twitter
23- Pinterest, Snapchat, Instagram, Twitter
24- YouTube, Facebook, Pinterest, Instagram
25- YouTube, Facebook, Pinterest, Snapchat
26- YouTube, Snapchat, Instagram, Twitter
27- Facebook, Snapchat, Instagram, Twitter
28- YouTube, Facebook, Snapchat, Instagram, Twitter
29- YouTube, Facebook, Pinterest, Snapchat, Instagram
30- YouTube, Pinterest, Snapchat, Instagram, Twitter
31- YouTube, Facebook, Pinterest, Snapchat, Instagram, Twitter

17. Attracted to food brand by watching Ad
    0- No
    1- Yes
    2- 2-Sometimes
18. Food Ads you come across

1. Chocolates
2. Chocolates, Fast Food chain
3. Fast Food chain
4. Fast Food chain, Food Delivery Platform
5. Fast Food chain, Food Delivery Platform, Chocolates
6. Fast Food chain, Food Delivery Platform, Chocolates, Fast Food chain
7. Fast Food chain, Food Delivery Platform, RTE foods, Chocolates
8. Fast Food chain, Food Delivery Platform, RTE foods, Chocolates, Chocolates
9. Fast Food chain, Food Delivery Platform, Snickers, Fast Food chain
10. Fast Food chain, Pizza chains
11. Fast Food chain, Pizza chains, Chocolates
12. Fast Food chain, Pizza chains, Chocolates, Chocolates
13. Fast Food chain, Pizza chains, Fast Food chain, Food Delivery Platform
14. Fast Food chain, Pizza chains, Fast Food chain, Food Delivery Platform, Chocolates
15. Fast Food chain, Pizza chains, Fast Food chain, Food Delivery Platform, RTE foods, Chocolates, Chocolates
16. Fast Food chain, Pizza chains, Fast Food chain, Food Delivery Platform, RTE foods, Chocolates, Snickers, Chocolates, Fast Food chain
17. Fast Food chain, Pizza chains, Fast Food chain, Food Delivery Platform, RTE foods, Fast Food chain
18. Fast Food chain, Pizza chains, Fast Food chain, Sandwich places, Food Delivery Platform, Chocolates, Fast Food chain

19. Fast Food chain, Pizza chains, Fast Food chain, Sandwich places, Food Delivery Platform, RTE foods, Chocolates, Snickers, Chocolates
20. Fast Food chain, Pizza chains, Food Delivery Platform
21. Fast Food chain, Pizza chains, Food Delivery Platform, Chocolates
22. Fast Food chain, Pizza chains, Food Delivery Platform, Chocolates, Fast Food chain
23. Fast Food chain, Pizza chains, Food Delivery Platform, RTE foods
24. Fast Food chain, Pizza chains, Food Delivery Platform, RTE foods, Chocolates
25. Fast Food chain, Pizza chains, Food Delivery Platform, RTE foods, Chocolates, Chocolates
26. Fast Food chain, Pizza chains, Food Delivery Platform, RTE foods, Chocolates, Snickers
27. Fast Food chain, Pizza chains, RTE foods
28. Fast Food chain, Pizza chains, RTE foods, Chocolates
29. Fast Food chain, Pizza chains, Sandwich places
30. Fast Food chain, Pizza chains, Sandwich places, Food Delivery Platform
31. Fast Food chain, Pizza chains, Sandwich places, Food Delivery Platform, RTE foods, Chocolates
32. Fast Food chain, Pizza chains, Sandwich places, Food Delivery Platform, RTE foods, Chocolates, Snickers, Chocolates, Fast Food chain
33. Fast Food chain, Pizza chains, Sandwich places, Snickers, Chocolates
34. Fast Food chain, RTE foods
35. Fast Food chain, RTE foods, Chocolates
36. Fast Food chain, Sandwich places
37. Fast Food chain, Sandwich places, Food Delivery Platform
38. Fast Food chain, Sandwich places, Food Delivery Platform, Chocolates
39. Food Delivery Platform
40. Food Delivery Platform, Chocolates
41. Food Delivery Platform, Chocolates, Fast Food chain
42. Food Delivery Platform, RTE foods
43. Food Delivery Platform, RTE foods, Chocolates
44. Food Delivery Platform, RTE foods, Chocolates, Chocolates
45. Food Delivery Platform, RTE foods, Chocolates, Snickers, Chocolates
46. Food Delivery Platform, RTE foods, Fast Food chain
47. NONE OF THE ABOVE
48. Pizza chains
49. Pizza chains, Chocolates
50. Pizza chains, Fast Food chain
51. Pizza chains, Fast Food chain, Food Delivery Platform
52. Pizza chains, Fast Food chain, Chocolates
53. Pizza chains, Fast Food chain, Chocolates, Chocolates
54. Pizza chains, Fast Food chain, Chocolates, Snickers
55. Pizza chains, Fast Food chain, Food Delivery Platform
56. Pizza chains, Fast Food chain, Food Delivery Platform, Chocolates
57. Pizza chains, Fast Food chain, Food Delivery Platform, Fast Food chain
58. Pizza chains, Fast Food chain, Food Delivery Platform, RTE foods
59. Pizza chains, Fast Food chain, Food Delivery Platform, RTE foods, Chocolates
60. Pizza chains, Fast Food chain, Food Delivery Platform, RTE foods, Chocolates, Snickers, Chocolates
61. Pizza chains, Fast Food chain, Pizza chains, Food Delivery Platform
62. Pizza chains, Fast Food chain, Pizza chains, Food Delivery Platform, RTE foods, Chocolates, Chocolates
63. Pizza chains, Fast Food chain, Pizza chains, Food Delivery Platform, RTE foods, Chocolates, Snickers, Chocolates
64. Pizza chains, Fast Food chain, Pizza chains, Food Delivery Platform, RTE foods, Chocolates, Snickers, Chocolates, Fast Food chain

65. Pizza chains, Fast Food chain, Pizza chains, Sandwich places, Food Delivery Platform, Chocolates, Chocolates
66. Pizza chains, Fast Food chain, Pizza chains, Sandwich places, Food Delivery Platform, Fast Food chain
67. Pizza chains, Fast Food chain, Sandwich places
68. Pizza chains, Fast Food chain, Sandwich places, Food Delivery Platform
69. Pizza chains, Fast Food chain, Sandwich places, Food Delivery Platform, Chocolates
70. Pizza chains, Fast Food chain, Sandwich places, Food Delivery Platform, RTE foods
71. Pizza chains, Fast Food chain, Sandwich places, Food Delivery Platform, RTE foods, Chocolates
72. Pizza chains, Fast Food chain, Sandwich places, Food Delivery Platform, Snickers
73. Pizza chains, Fast Food chain, Sandwich places, RTE foods
74. Pizza chains, Fast Food chain, Sandwich places, RTE foods, Chocolates
75. Pizza chains, Fast Food chain,, Food Delivery Platform
76. Pizza chains, Fast Food chain,, Food Delivery Platform, Fast Food chain
77. Pizza chains, Fast Food chain,, Food Delivery Platform, RTE foods
78. Pizza chains, Fast Food chain,, Food Delivery Platform, RTE foods, Chocolates
79. Pizza chains, Fast Food chain,, Food Delivery Platform, RTE foods, Chocolates, Chocolates, Fast Food chain
80. Pizza chains, Fast Food chain,, Food Delivery Platform, RTE foods, Chocolates, Snickers, Chocolates, Fast Food chain
81. Pizza chains, Fast Food chain,, Food Delivery Platform, RTE foods, Fast Food chain
82. Pizza chains, Fast Food chain,, Sandwich places, Chocolates, Chocolates
83. Pizza chains, Fast Food chain,, Sandwich places, Food Delivery Platform, Chocolates
84. Pizza chains, Fast Food chain,, Sandwich places, Food Delivery Platform, RTE foods, Chocolates, Chocolates, Fast Food chain
85. Pizza chains, Fast Food chain,, Sandwich places, Food Delivery Platform, RTE foods, Chocolates, Snickers, Chocolates
86. Pizza chains, Fast Food chain,, Sandwich places, Food Delivery Platform, RTE foods, Chocolates, Snickers, Chocolates, Fast Food chain
87. Pizza chains, Fast Food chain,, Sandwich places, Food Delivery Platform, RTE foods, Chocolates, Snickers, Chocolates, Fast Food chain, NONE OF THE ABOVE
88. Pizza chains, Fast Food chain,, Sandwich places, Food Delivery Platform, RTE foods, Fast Food chain
89. Pizza chains, Fast Food chain,, Sandwich places, Food Delivery Platform, RTE foods, Snickers, Chocolates
90. Pizza chains, Fast Food chains, Food Delivery Platform, RTE foods, Chocolates
91. Pizza chains, Food Delivery Platform
92. Pizza chains, Food Delivery Platform, Chocolates
93. Pizza chains, Food Delivery Platform, Chocolates, Chocolates
94. Pizza chains, Food Delivery Platform, Fast Food chain
95. Pizza chains, Food Delivery Platform, RTE foods
96. Pizza chains, Food Delivery Platform, RTE foods, Chocolates
97. Pizza chains, Food Delivery Platform, RTE foods, Chocolates, Chocolates
98. Pizza chains, Food Delivery Platform, RTE foods, Chocolates, Fast Food chain
99. Pizza chains, Food Delivery Platform, RTE foods, Fast Food chain
100. Pizza chains, RTE foods
101. Pizza chains, RTE foods, Chocolates
102. Pizza chains, RTE foods, Chocolates, Snickers, Chocolates
103. Pizza chains, Sandwich places
104. Pizza chains, Sandwich places, Chocolates
105. Pizza chains, Sandwich places, Food Delivery Platform, Fast Food chain
106. RTE foods
107. RTE foods, Chocolates
108. Sandwich places, Food Delivery Platform

109. Sandwich places, Food Delivery Platform, Fast Food chain
110. Sandwich places, Food Delivery Platform, RTE foods

19. How often do you see food content and ads on the social media you use?
    0- Never,
    1- Once a week,
    2- Everyday
    3- After every few minutes
    4- After every few posts and stories
20. Do you love watching food ads and content?
    0- No
    1- Yes
21. How likely are you to try a food/food product/Restaurant/ Eatery promoted by a social media influencer?
    0- Never
    1- Rarely
    2- Occasionaly
    3- Frequently
    4- Very frequently
22. Do you like to view food pictures others post on social media?
    0- No,
    1- Yes,
    2- Sometimes
23. Do you order and eat something you see in a food ad/content when you are stressed/emotional?
    0- No
    1- Yes

24. Visual Analog Scale

1= Low

2= Moderate

3= High
